# A Pilot Trial of a Manualized Psychoeducation Module for Parents of Children with Autism with Intellectual Disability and Intellectual Disability Alone

**Published:** 2020-09-28

**Authors:** Nupur Kumari, Triptish Bhatia, Satabdi Chakraborty, Anuradha Balsavar, Smita N. Deshpande

**Affiliations:** Department of Psychiatry and De-addiction, Centre of Excellence in Mental Health, ABVIMS - Dr. Ram Manohar Lohia Hospital, New Delhi, India; 1 Department of Psychiatry, ATC-Central Council of Research in Ayurvedic Sciences, Dept. of Psychiatry, PGIMER- Dr. R.M.L Hospital, New Delhi, India

**Keywords:** Autism, developmental disorders, intellectual disability, Indian Scale for Assessment of Autism, module, psychoeducation

## Abstract

**Objectives:**

Children with autism with/without intellectual disability (ID) and ID alone require regular interventions. Psychoeducation (PE) can empower parents with intervention strategies. The aim of this study was to develop the test efficacy of a simple, short manualized PE module for parents of children with autism with/without comorbid ID and for ID alone. We focused on both autism and ID (A-ID) because we felt that both the groups could benefit from this module.

**Methods:**

A special module for PE was developed after literature review, inputs from a study group, and discussion with experts. Parents attended eight fortnightly intervention sessions. Children were assessed on the Developmental Screening Test, Indian Scale for Assessment of Autism (ISAA), and the Behavioral Assessment Scale for Indian Children with Mental Retardation (BASIC-MR) before starting and 1 month after completing PE.

**Results:**

Consenting parent of parents/of 16 children with A-ID and 14 with ID completed sessions with pre- and postassessment. There was a significant improvement in the majority of domains of ISAA and BASIC-MR Part B in children with both conditions.

**Conclusions:**

PE has a wide scope for use across various developmental disorders. The module developed is promising for a wide variety of field workers.

## Introduction

Autism spectrum disorder (ASD) is a chronic neurodevelopmental condition of early childhood onset characterized by social communication deficits and restricted and repetitive patterns of behavior.^[1]^ Intellectual disability (ID) (previously termed mental retardation [MR]) is a disorder with onset during the developmental period that includes both intellectual and adaptive functioning deficits in conceptual, social, and practical domains.^[1]^ The two may occur independently or together. Several studies have reported varying but significantly high rates of comorbidity in persons with autism/ID.^[2]^ Autism was associated with MR in about 70% of cases and was more than four times likelier to occur in males than in females.^[3]^ Up to 40% of the ID population may meet the diagnostic criteria for ASD.^[4,5]^ Among Indian children with ID, autism was diagnosed in 23.6% with a male: female ratio of 3.1:1.^[6]^ In a report from the Centers for Disease Control and Prevention, the United States, 31.6% of children with autism had comorbid ID, with a male–female ratio of 4.3:1.^[7]^ Differential recognition of autistic symptoms in children with ID^[2,8–10]^ may lead to these varying rates. Some autism symptoms, such as impaired communication and hyperactivity, may overlap with symptoms of ID. Symptoms of autism may be the main vulnerability factor for increased psychopathology among ID populations.^[11,12]^ Other disorders may also be commonly prevalent. A study from Bangalore, India,^[13]^ reported 46% psychiatric comorbidity in children with ASD; the most comorbidity attention deficit hyperactivity disorder. Children with autism present with behavioral problems including temper tantrums, noncompliance, aggression, and self-injurious behavior.^[14,15]^ These affect their skills of independent living and increase social isolation.

Due to a lack of awareness and the absence of available services in India, Singh *et al*. reported many problems and unmet needs among parents of children with autism. Mothers of children with autism suffer high rates of depression and burden, partly mitigated by social support (which is often lacking).^[16]^ Parents of children with autism and ID (A-ID), versus parents of children with ID alone, experience similar rates of burden.^[17]^ Parental training may bridge this gap.^[18]^ Parental involvement in their child’s training has long been regarded as an important component of early intervention programs for children with autism.^[19]^ The possible benefits of parental training are increased skills and confidence (of children) and reduced stress for parents and children.^[20,21]^ Malhotra emphasized the usefulness of parental training for interventions for behavioral issues of their children in Indian settings.^[22]^ Intervention in ASD can be carried out by the combined effort of a multidisciplinary team and mostly consists of behavioral modification strategies,^[23]^ since parents are the primary caregivers; behavioral problems of their child affect them the most. Changes in the parent’s handling of the child help improve symptoms in the child as well. A Cochrane review^[24]^ and randomized controlled trial^[25]^ in autism provide evidence suggesting that parents can be successfully educated to train their children, especially on communication and social skills. Parenting interventions can reduce child problem behavior and improve desired behavior.^[26]^

Different methods have been used for parental training. In North India, Patra developed a parental psychoeducation (PE) module and tested it in a pilot intervention study to evaluate its effect on parental stress and knowledge.^[27]^ A recent study used WhatsApp.^[28]^ They used one face-to-face session (60 min) and four virtual sessions (30 min each), with parental psychological issues being the primary outcome and child behavior problems and ASD symptoms being the secondary ones. Parental training was compared to parental PE in a randomized control trial for parental stress, strain, and competence.^[29]^ Successful parental PE interventions include the Preschool Autism Communication Trial (PACT), the largest randomized clinical trial (RCT) on autism by the Medical Research Council, Britain, with the outcome being improvement in day-to-day basic skills of the child.^[30]^ Green *et al*. in their multicentric RCT are testing PACT-G intervention in autism symptoms in the home and research settings using a standardized protocol.^[31]^ A mirror study in South Asia – termed the parent-mediated intervention for ASD in South Asia – also reported positive effects of parent-initiated intervention.^[32]^ Communication-centered parent-mediated treatment for ASD in South Asia, Autism Distance Education Parent Training, is ongoing, but results are not yet available.^[33]^

The above-quoted studies focused mostly on communication. Considering the socioeconomic and educational levels of parents commonly availing of free government facilities such as ours, we decided to focus on troublesome symptoms and individual development as a whole. It was also necessary to develop as brief a module as possible, as parents needed to miss 1 day’s wages to attend sessions. The goal was to train the parents themselves to deal with behavioral and symptomatic problems in their children, which they faced day after day. Thus, the present pilot trial was undertaken to test the efficacy of a specially developed PE module for parental training for improving behavior for children with A-ID and for those with ID alone.

## Methods

As a part of a larger research project (Ref no. DST No.: SR/CSI/30/2008). a PE module was developed, and parents and children with autism with/without ID and ID alone were recruited. While the first two objectives were genetic and ethical, parents who agreed to participate were to be offered a specially developed, simple-to-implement relatively brief, and free-to-use PE program to help them tackle day-to-day issues around their child’s condition. The module was developed with the objective to help parents of children with autism with/without ID and ID alone in supporting their children to improve autistic symptoms and behavioral problems. The program was tested on participants who consented to participate. The program was individualized as different problems are faced by these children and with differing severity.

The efficacy of the module was evaluated through actual change in behavior among children rather than its effect on parental stress or symptoms. The study was conducted from August 2012 to January 2015 at the Department of Psychiatry, PGIMER-Dr. RML Hospital, New Delhi, a tertiary care postgraduate government teaching hospital offering free services. Ethical permission was obtained from the Institutional Ethics Committee of this institution.

### Psychoeducation module

Studies have reported that it is difficult for children with autism to generalize acquired knowledge.^[34]^ Social skills of children with autism can be improved.^[35]^ Naturalistic learning by parents helping the child in their natural environment is the best practice for intervention.^[19]^ The Family-Implemented Treatment for Behavioral Inflexibility successfully targeted repetitive behavior in autism.^[36]^

We developed a PE module for parents of children with A-ID as follows:

#### Collection of study material

PubMed, Google Scholar, and PsycINFO were searched using search words “autism and psychoeducation,” “autism and teaching,” and “autism and learning,” “activities of daily living and autism,” and “skills training in autism.” Relevant research articles in the field were compiled and studied. Educational and research material related to A-ID was collected from specific websites including the Action for Autism, National Institute for the Empowerment of Persons with IDs, National Centre for Autism, and others. We could not find a comprehensive module of PE in Indian database.

#### Study group

A study group was formed comprising psychologists, psychiatric social workers, and psychiatrists working with children. The needs and requirements of families with children with ID/autism were studied extensively before the group designed the basic draft. It needed to be simple and generalizable to families of all educational and socioeconomic strata and useful for both children with A-ID.

#### Expert group consultation

A second expert group of specialists specifically working in the field of A-ID, independent of the first group, was approached, and the draft module was discussed with them. These experts were working with children of varied social strata. They had extensive experience with parents, their questions, needs, and worries. The first draft was modified after considering expert comments. Experts opined that parents needed guidance and education and that most would be willing to attend PE sessions in order to take over the training of their children in their own hands.

### The final module

The final module, capable of being individualized according to the needs of children with autism and of children with ID, comprised eight fortnightly sessions (4 months) for individual parent participation. It addressed areas of education about illness, family support, crisis intervention, and also contained problem-solving skill training. The aim of the intervention was to increase parental sensitivity and responsiveness to child communication and reduce mistimed parental responses by working with the parent. A parent was instructed to continue to follow the techniques at home after the intervention. Between sessions, a parent was also asked to undertake regular 30 min of daily home practice. A description of the module is presented below:

#### Session 1

##### Parental education

The first session provided information about the illness and its effect on the child’s emotions and behavior. The symptoms and problems relating to autism and/or ID were discussed. Causes of the condition as well as prevalence were discussed. This helped parents to modify their expectations and emphasized their importance in training. A parent was made aware of specific needs, strengths, and weaknesses of their child. Lack of parents’ awareness and training leads to parental stress and burden,^[37]^ and educating them can decrease the stigma as well as increase the motivation to help the child.^[27]^

#### Session 2

##### Improve eye contact

After making the parents aware of the whole picture of the illness in the first session, the second session started with specific symptoms which were worrying to the parents. Eye contact is an important feature of social communication, and children with autism frequently lack eye contact. Mand responses (reinforcement) can improve behavior in terms of eye contact.^[38]^ Hence, the second session focused on improving eye contact through methods including using an overly enthusiastic voice and holding objects close to one’s own eyes and then waiting for eye contact before complying with the child’s requests. A parent was instructed to hold the child’s face while talking. For sustained eye contact, reinforcement was increased and the target for duration of eye contact was also increased after attainment of the first target, for example, if first the target was set at 5 s, the interval was progressively prolonged. The number of seconds passed was counted while the child made eye contact.

#### Session 3 and 4

##### Communication

The next two sessions (third and fourth) were based on communication and holistic learning of language. Pickles *et al*. reported that long-term intervention (1 year) improves the communication skills of children with autism.^[30]^ Hence, if parents, who are permanently present, are trained to teach communication skills, it will be more advantageous for their child, for example, the use of exaggerated prosody and repetitive paraphrasing to maximize the likelihood of the child understanding the meaning of key referent words. In these sessions, a parent was trained to use visual support and simple gestures for spoken language (objects, photos, and pictures). They were told to call the child by name, encourage them to talk while playing, encourage them to participate in turn-taking games, and talk to them while playing. A parent was asked to choose toys with lights and sounds. They were suggested to engage children in physical activity to develop fine motor skills and use reinforcement every time the child showed desirable behavior.

#### Session 5

##### Object and color identification

In the fifth session, object and color identification was discussed using blocks and balls of different colors. A parent was asked to repeat the task many times and gradually introduce more colors. A parent showed the pictures of common household objects, said the name of the object, and asked the child to point it out when asked. A parent taught the child counting with beads and reinforcement. A parent was instructed not to introduce more than one color or object at a time. When children started recognizing a particular color or object only then, introduce the second one. Parents should repeat every learned item after short intervals so that child keeps remembering. This module was specifically included as our expert panel suggested it, as also literature. For instance, Ludlow *et al*.^[39]^ suggested that children with autism have color obsessions and phobias which need to be addressed. Colors presented with cues were easier to learn than without.^[40]^

#### Sessions 6 and 7

##### Behavior modification

The next two sessions (sixth and seventh) were focused on behavior management techniques including principles of reinforcement, interrupting unwanted behavior, and teaching alternative behaviors. The parent was advised to assess problem behavior and focus on rectifying that behavior only. A hierarchy of problem behaviors was made, and each behavior targeted one by one with reinforcement. When a child showed problem behavior, an attempt was made to shift his/her attention to something that interested him/her. It is very important to manage behavioral problems in the comprehensive rehabilitation of people with ID as well as autism.^[41]^ Autistic children also show several behavioral problems, and these can be rectified with reinforcement and teaching.

#### Final session (8)

##### Self-help

The last session of the training was designed to develop self-help skills. There were applied behavior analytic (ABA) methodologies for teaching a variety of skills that produce lasting functional improvements in many children with autism.^[42]^ Parents need to be educated in these strategies and can modify the symptoms of their children. A parent was taught to break each task into small steps and not to move ahead unless the first task was mastered. Each step was to be reinforced. A parent was invited to discuss any problems they experienced between sessions and also asked to do 30 min of daily home practice.

The content of each session of the module was additionally individualized according to the needs of the family and its cultural, social, and educational background. Although the overall structure remained the same, a parent was encouraged to focus on the child’s specific problem/s. More time was devoted to resolve the specific issues in that particular session. A parent was instructed to follow the techniques at home. Either the mother, the father, or a constant caregiver could train the child. The average time taken was approximately 1–1.5 h for the initial session and 45 min to 1 h for each subsequent session. The sessions were used to train the parents, and then, they had to practice at home. In all subsequent sessions, the learnings of previous sessions were repeated in brief so that their queries, if any, could be addressed.

##### Prepilot testing

The module was tested on ten parents of children with autism and modified using suggestions and expressed needs of the parents.

The entire module, along with frequently asked questions by parents, is included as a supplement.

### Participants

Children diagnosed with autism with/without ID and ID alone along with their accompanying parents presenting in OPD of the department of psychiatry of a tertiary care hospital were informed about the study by their treating psychiatrists. After oral consent, they were referred to the research team who obtained written informed consent after explaining study procedures in detail. A parent could consent for all or any or none of the research project objectives. The inclusion criteria were children aged 3–18 years scoring 70 and above on the Indian Scale for Assessment of Autism (ISAA) (for autism) and/or with moderate-to-severe levels of ID on a reliable intelligence test (for ID). Those who provided written informed consent (parents) and assent (eligible children) were included.

One parent of all consenting participants attended eight fortnightly psychoeducational intervention sessions. Qualified clinical psychologists or psychiatric social workers imparted PE after due training. Participants were assessed on the following assessment instruments at baseline and after completion of PE. Parents came for eight fortnightly intervention sessions (i.e., two sessions per month). Thus, the entire module took a minimum of 4 months, and postassessment was carried out 1 month after the last session. We measured the developmental quotient of the children. The mean development quotient of children with/without autism was 50.19 ± 22.9 and children with ID alone was 44.28 ± 15.57.

### Assessment instruments

#### Indian Scale for Assessment of Autism

The ISAA^[43,44]^ is a 40-item scale divided into six domains – Social Relationship and Reciprocity, Emotional Responsiveness, Speech-Language and Communication, Behavior Patterns, Sensory Aspects, and Cognitive Component. The scoring for each item of ISAA ranges from 1 to 5 depending on the intensity, frequency, and duration of a particular behavior. The total ISAA scores range from 40 to 200. The categories of ISAA are as follows: no autism: 69 and below, mild autism: 70–107, moderate autism: 108–153, and severe autism: 153 and above. Criterion test validity of ISAA was obtained by comparing ISAA with CARS using Pearson’s product moment correlation which is *r* = 0.77 (*P* < 0.001). The reliability of ISAA is 0.93 (*P* < 0.001).

In our earlier study, ISAA scores in children with ID were significantly lower than those in children with autism.[45,3] Scoring sheet of ISAA is attached as a supplement. The ISAA is now the legal instrument for certifying disability for children with autism under the Indian law.

#### Behavioral Assessment Scale for Indian Children with Mental Retardation Part B

The Behavioral Assessment Scale for Indian Children with MR (BASIC-MR) Part B^[45]^ has a total of 75 items, covering 10 domains. For any given child with “mental handicap,” each item of the scale is checked and rated on a three-point scale, namely never, occasionally, and frequently. Each item score ranges from 0 to 2 depending on the severity and frequency of that problem behavior. Its test–retest reliability coefficient is 0.68 and its construct validity is statistically significant (*P* ≤ 0.001).

#### Developmental Screening Tes

This test has a simplified version for the age range of 3 months to 15 years.^[46]^ It consists of 88 items that represent the behavioral characteristics of the respective age levels. Appraisal of a child can be done through a semi-structured interview with a parent or a person well acquainted with the child. The IQ calculator incorporated in the test folder helps in ready computation of IQ from the mental age and the chronological age of the child. Inter-scorer reliability and test–retest reliability of the test are 0.928 and 0.98, respectively.

### Administration

The module was administered by qualified mental health professionals – either clinical psychologists or psychiatric social workers with at least with MPhil degree from a recognized institution. These were full-time research personnel who were trained in the implementation of the module through teaching and observation. Assessment was carried out by a research staff who had not administered the module. Postassessment was carried out 1 month after completion of intervention.

### Data analysis

Demographic and clinical variables were compared using the Mann–Whitney U-test after checking the normality of the data. The effect sizes were calculated separately for both the groups using Cohen’s D. The Wilcoxon signed-rank test was used to assess the significance of change as sample was small and not normal. We used IBM SPSS Statistics for Windows, Version 23.0. for the data analysis.^[47]^

## Results

### Dempgraphic details of psychoeducation participants (comparison of the completers vs. noncompleters)

A total of 100 participants (A-ID *n* = 55 and ID alone *n* = 45) agreed to participate. We excluded seven ID participants because of their level of severity and four high functioning children with autism because their IQ was within normal range, and there were no comparable participants in the ID population. We excluded 6 participants with incomplete assessment due to the parents’ busy schedule and/or the children’s inability to cooperate. Hence, 83 participants were considered for baseline analysis (A-ID, *n* = 45 [34 M/11 F], and ID alone, *n* = 38 (29 M/9 F). They were assessed before PE and were asked to come for PE sessions. Many were staying far away so could not start PE. Those who started could not complete all sessions due to commuting expenses or job duties. Finally, one parent each, of 16 children with A-ID and 14 children with ID alone, completed all procedures of PE and postassessment [Table 1].

### Baseline comparison of children who did or did not complete the study

We recruited a large number of participants at baseline, but many dropped out after that or during PE. Reasons for dropout among others were living far away, commuting expenses, other children to look after. We compared participants who dropped out and did not complete the study with those who completed all PE modules. There was no significant difference between completers and noncompleters on any of the demographic variables [Table 1]. There was no significant difference on ISAA domains between completers and noncompleters. However, on BASIC-MR antisocial behavior subscale, completers scored higher on repetitive behavior, self-injurious behavior, and temper tantrums.

### Demographic and baseline comparison of the participants on the Indian Scale for Assessment of Autism and Behavioral Assessment Scale for Indian Children with Mental Retardation subscales

We compared ISAA scores, total score, and domain scores at baseline. All children were also administered BASIC-MR Part B scale at baseline. There was no significant difference between the two groups on any of the demographic variables [Table 1] except that the children with ID were older than children with A-ID. Majority of the parents in both the groups were skilled workers and belonged to low socioeconomic status as indicated by their monthly salary. As expected, both the groups were significantly different on all domains of the ISAA [Table 2]. There was no significant difference between children with A-ID and ID alone on BASIC-MR subscales.

We then compared the baseline ISAA and BASIC-MR scores of completers. On all ISAA domains and total ISAA scores (*P* < 0.000001), A-ID scored higher than those of ID children. There was no age difference between the two groups [Table 2]. There was no significant difference between the two groups on any subscales of BASIC-MR Part B. On GAF scores, also both the groups were similar [Table 2].

### Change in the Indian Scale for Assessment of Autism and Behavioral Assessment Scale for Indian Children with Mental Retardation Part B scores after intervention

A significant improvement was observed in ISAA after PE in the A-ID group in social relationships and reciprocity (*P* = 0.002), emotional responsiveness (*P* = 0.03), speech and communication (*P* = 0.01), and total ISAA score (*P* = 0.001) [Table 3]. GAF scores also improved after PE (*P* = 0.004). In the same group, a significant improvement was also observed in various domains of violent behavior (*P* = 0.019), hyperactivity (*P* = 0.038), and total BASIC-MR Part B score (*P* = 0.05) [Table 3]. ISAA was not administered on the ID group. However, on BASIC-MR subscales, a significant improvement was there on certain items: violent behavior (0.06), self-injurious behavior (0.02), repetitive behavior (0.024), and odd behavior (0.03) decreased significantly after PE. Total BASIC-MR scores also decreased suggesting improvement in behavior (0.01) [Table 3].

## Discussion

Children with autism and/or ID have differing neurodevelopmental disabilities. However, they may nevertheless share similar symptoms such as early childhood developmental delays, limited speech and vocabulary, problems understanding verbal instructions and following directions, difficulty communicating with peers, need of life skill training, phrases out of context, and repetitive behavior. Therefore, we decided to test whether similar PE training could benefit both the groups with respect to similar symptoms. The module developed tried to touch upon all similar aspects of behavioral problems in A-ID. Each session was thoughtfully conceptualized. The principles of working with families were considered while designing the module.^[48,49]^

Visits to the hospital, once in 15 days, was considered to be an appropriate interval for sessions to give time to parents to test the training in the previous session. If sessions were too wide apart, parents could forget many instructions. The individualized training sessions, once a fortnight, provided enough flexibility.

The diagnosis of autism was based on ISAA scores. A child/adolescent with a score of 70 and above was considered to be having autism. Scores on IQ tests were taken as a secondary diagnosis (A-ID). A child/adolescent with scores on the relevant standardized IQ tests in the moderate or severe range was eligible (A-ID or ID alone) as we wanted to enroll a clinically representative sample. We could enroll only four high functioning children with autism alone. Thus, a majority of the children enrolled had autism with ID in accordance with earlier studies which state that between 40% and 70% of children with pervasive developmental disorders have co-occurring ID.^[3,43,50,51]^ The prevalence of ID in the general population is 1%, and 10% among them may have ASD or autistic traits, while 38% of individuals with ASD may have ID.^[52]^

Males were overrepresented (75% among A-ID and 72% among ID alone) as in earlier studies.^[3,53]^ The higher number of males in our ID sample may also be because of social reasons. Since Indian society is more concerned about their male children, the manifestation of any disability in their children is reported more among male children than female ones.^[43]^

Parents of children with A-ID observed significantly more temper tantrums, repetitive behavior, odd behavior and hyperactivity than those of ID alone. However, they reported same degree of violent, self-injurious, rebellious, antisocial behaviors and fear. Other studies have also reported higher hyperactivity in children with ASD.^[54]^ Repetitive behavior and temper tantrums were reported significantly more in children with autism than in those with ID.^[55,56]^ Aggression (violent behavior, rebellious behavior, and self-injury) is reported in ASD,^[57,58]^ but this behavior was observed in our ID sample also. Individuals with comorbid autism with ID report higher levels of self-injury than individuals without ID.^[59,60]^

Most of the parents in our study had very limited information about the condition their child suffered from. Only the very educated were knowledgeable about ASD. This is true of Indian parents who ascribe their child’s condition to the “will of God” or “a test by God.”^[61]^ It is even more critical to design intervention programs for children with comorbid ASD with ID. Patra *et al*.^[27]^ developed a PE module, especially for children with autism testing the stress and knowledge of parents before and after PE and reported improvement. However, this group did not focus on changes in the child’s behavior. Minhas *et al*. suggested that motivated family members could be trained in recognizing and providing evidence-based interventions as they are burdened and under stress because of their children’s behavior.^[61]^ In the current study, we wanted to examine if parental PE intervention could successfully modify the child’s behavior. The intervention is specifically designed to be individualized as the heterogeneity and developmental nature of the disorder make it unlikely that one specific treatment will be best for all children with autism.^[62]^ Due to various constraints, parental stress or other parental measures could not be assessed. We planned to develop a short yet effective module, since existing services were either expensive, distant, or required long periods of parental involvement. The PE module addressed parental concerns and helped them modify their child’s symptoms. After intervention, several symptoms were successfully altered. However, in spite of developing a relatively brief intervention, parents could not find time to attend. Hence, they either dropped out or did not participate at all. Larger field trials can help in examining specific tasks and their duration of learning, however, the learning also depends on individual characteristics of parents and children. The individualized program can help in identifying the tasks which need more sessions and which need single sessions.

While we found the PE intervention relatively successful and acceptable, others have not. Iadarola *et al*.^[29]^ found parental training more effective in reducing parental stress. Shire *et al*.^[63]^ found their Joint Attention, Symbolic Play, Engagement, and Regulation system better than PE in improving parental responsively and joint engagement of the child for social communication and play skills. They did not address problem behavior exhibited by the participants as in the current study. Parents who did complete participation observed several changes in the child’s behavior over the period of the study. Core symptoms, such as social relations, communication, and emotional responsiveness, improved significantly by the end of the study. Symptoms, such as violence, temper tantrums, and even hyperactivity, improved significantly. No behavioral change was reported in children with ID.

Empowering the parent and teaching them behavior modification skills helped the child as well. Investigators in Saudi Arabia reported a reduction in stress and depression in mothers with children with autism, with only one face to face session, followed by four WhatsApp sessions.^[28]^ Positive change persisted for 8 weeks. However, a second group found that training parents in behavioral strategies helped to reduce their stress and improved their sense of competence, while PE alone was less effective.^[29]^ Since our module incorporated some behavioral strategies as well, this module may have been more effective in changing the child’s behavior.

## Limitations

Participant parents accepted the PE module in the initial stage and completed the baseline assessment enthusiastically. They agreed to come for all sessions initially but could eventually not come, possibly because of nihilism, lack of time, or loss of wages although a small travel allowance was paid for attendance. Some dropped out after one or two sessions. Only one-third of the sample completed postintervention assessment. The high attrition may be attributed to the fact that most of the participants came from lower socioeconomic status who worked as daily wage laborers far from the hospitals and were unable to afford the commute to the hospitals. They had to take leave which also was an issue. The caregivers were not educated also which resulted in caregivers expressing a challenge in translating PE learning in the home environment and hence did not feel motivated to continue. Many of these parents reported that their children attended special schools, and some reported that they were unable to take leave from their work. Thus, evening or weekend sessions or home-based interventions may be needed for a wider reach. Group interventions may also be more effective (such programs have been described for adolescents and older people with autism).^[64,65]^ Some parents may need repeated sessions, perhaps spread over a year or more, as and when needed/ required. One session per issue may not be sufficient to train parents adequately. However, they could be asked to come again to clarify doubts.

Other limitations are the small comparison sample size and lack of adequate number of children with autism alone. Furthermore, effect sizes were possibly high because highly motivated parents completed all the sessions and completed postassessment. The follow-up was done after 1 month after completion of intervention. A much longer follow-up, along with measurable changes on other specific measures, would also have strengthened our results but could not be done due to logistic constraints.

## Strengths and implications

Developing a written, detailed protocol was one of the strengths of this study as it could be used to train other mental health professionals. Lack of detailed intervention and implementation were lacunae pointed out in a systematic review of early intensive behavioral intervention programs for children with autism.^[66]^ Such brief, protocolized programs can be easily administered even in schools with the help of psychologists and counselors ensuring individual training.

## Conclusions

We describe the steps followed for developing a step-by-step PE intervention module for parents of children with comorbid A-ID. The module consisted of 8 fortnightly sessions of approximately 1 h. The relatively fewer sessions were designed for parents with less time and resources for their affected child, as well as the lack of affordable services for such children. Considering the behavioral issues of greatest distress to the parents, we focused on child’s symptoms and their modification. We can test this manualized module with a field trial including an adequately powered bigger sample, pre- and postevaluation of specific targeted tasks. The follow-up should be of even longer period, so that long-term effect of the intervention is retained. We would also like to train persons with MA degree to see if task shifting can be successful so as to reach a larger population.

## Figures and Tables

**Table 1:** Comparison of sociodemographic variables between the completers (*n*=30) and noncompleters (*n*=53) of psychoeducation modules

Sociodemographic variable	Noncompleters (*n*=53)	Completers (*n*=30)	*χ*^2^/*Z*	*P*

Age (months)	106.47±49.22	93.00±39.88	−0.860	0.39
Sex, male/female	42/11	21/9	0.895	0.43
Diagnosis, A-ID/ID	29/24	16/14	0.014	1.00
Father’s literacy, 1/2/3/4/5*	3/2/6/16/26	2/1/4/7	8.15	0.08
Mother’s literacy, 1/2/3/4/5*	5/4/8/13/23	8/5/3/7/7	2.45	0.23
Parents’ occupation, 1/2/3/4**	1/6/33/13	0/4/18/8	2.08	0.57
Family monthly income, 1/2/3/4***	3/27/13/10	3/18/5/4	5.38	0.53

**ISAA scores: No significant difference on ISAA domains was found between completers and noncompleters (data available with authors)**				

Past month GAF	42.17±10.82	38.33±13.13	−1.367	0.17
BASIC-MR Part B subscales		Significantly different variables only		
Repetitive behavior	4.57±4.46	7.23±5.81	−2.206	0.03
Self-injurious behavior	4.19±5.24	6.60±6.40	−2.128	0.03
Temper tantrums	4.06±3.68	6.37±3.77	−2.580	<0.01
Total score	40.38±30.12	55.60±30.34	−2.389	0.01

* Illiterate/read and write/nonmatriculation/matriculation/graduation and above

** Unemployed/field work/skilled/unskilled

*** Below 5000/5000–20,000/20,001–50,000/50,001 and above.

BASIC-MR=Behavioral Assessment Scale for Indian Children with Mental Retardation, GAF=Global Assessment of Functioning, ISAA=Indian Scale for Assessment of Autism

**Table 2:** Comparison of baseline Indian Scale for Assessment of Autism and Behavioral Assessment Scale for Indian Children with Mental Retardation subscales/domains between completer children of autism and intellectual disability and intellectual disability alone

ISAA subscales	Mean±SD		*Z*	*P*
	A-ID (*n*=16)	ID (*n*=14)		

Age (months)	84.00±32.63	103.29±45.89	−1.027	0.31
Social relationship	35.31±6.75	13.93±3.05	−4.664	<0.001*
Emotional responsiveness	14.06±3.51	9.03±3.27	−3.135	0.002*
Speech and communication	25.56±8.91	12.36±2.24	−4.165	<0.001*
Behavior pattern	21.31±4.67	12.86±2.38	−4.174	<0.001*
Sensory aspects	11.75±3.454	8.21±2.26	−2.823	<0.004*
Cognitive component	8.88±2.527	6.21±1.19	−2.983	0.002*
Total ISAA score	116.88±21.02	62.86±6.90	−4.663	<0.001*
Past month GAF	34.31±15.26	42.93±8.54	−1.727	0.08
BASIC-MR Part B subscales				
Violent behavior	11.13±8.065	17.14±11.35	−1.457	0.15
Temper tantrum	6.38±3.557	6.36±4.144	−0.021	0.99
Misbehavior	5.19±4.520	5.50±5.75	−.0168	0.88
Self-injurious behavior	6.06±5.916	7.21±7.09	−0.563	0.59
Repetitive behavior	8.50±5.441	5.79±6.08	−1.462	0.15
Odd behavior	6.31±4.27	8.29±6.44	−0.710	0.49
Hyperactivity	6.25±2.93	4.29±3.31	−1.787	0.08
Rebellious behavior	1.13±1.59	2.00±2.45	−1.103	0.29
Antisocial behavior	0.38±1.025	0.71±1.44	−0.706	0.52
Fear	1.88±2.75	1.07±1.44	−0.353	0.74
Total score	53.19±26.235	58.36±35.27	−0.416	0.69

* *P*<0.005 statistically significant.

BASIC-MR=Behavioral Assessment Scale for Indian Children with Mental Retardation, ISAA=Indian Scale for Assessment of Autism, SD=Standard deviation, A-ID=Autism and intellectual disability, ID=Intellectual disability

**Table 3:** Improvement among the Indian Scale for Assessment of Autism profile and Behavioral Assessment Scale for Indian Children with Mental Retardation Part B score among completer groups: autism and intellectual disability (*n*=16) and intellectual disability (*n*=14) (significant changes only)

ISAA (administered to A-ID alone)	Mean±SD		Effect size Glass’s delta	Cohen’s *D*	Wilcoxon *Z*	*P*
	Preassessment	Postassessment				

Subscales (A-ID)						
Social relationship	35.31±6.75	29.13±9.12	−0.92	−0.77	−3.11	0.001
Emotional responsiveness	14.06±3.51	11.06±4.46	−0.85	−0.74	−2.22	0.03
Behavior pattern	21.31±4.67	16.63±5.07	−1.00	−0.96	−2.45	0.01
Total ISAA score	116.88±21.02	96.69±22.79	−0.96	−0.92	−2.56	0.01
Past month GAF	34.31±15.27	41.13±14.61	0.45	−0.46	−2.85	0.004
BASIC-MR Part B subscales (A-ID)						
Violent behavior	11.13±8.07	6.69±5.52	−0.55	−0.64	−2.355	0.02
Hyperactivity	6.25±2.93	5.06±2.77	−0.41	−0.42	−2.198	0.03
Total score	53.19±26.24	39.50±23.78	−0.52	−0.55	−1.957	0.05
BASIC-MR Part B subscales (ID)						
Violent behavior	17.14±11.35	12.21±13.89	−0.43	−0.32	−2.091	0.04
Self-injurious behavior	7.21±7.11	5.07±6.51	−0.30	−0.31	−2.427	0.02
Repetitive behavior	5.79±6.08	2.71±3.51	−0.51	−0.62	−2.252	0.02
Odd behavior	8.29±6.44	6.29±6.46	−0.31	−0.31	−2.207	0.03
Total score	58.36±35.27	41.43±39.74	−0.48	−0.45	−2.535	0.01

BASIC-MR=Behavioral Assessment Scale for Indian Children with Mental Retardation, ISAA=Indian Scale for Assessment of Autism, SD=Standard deviation, A-ID=Autism and intellectual disability, ID=Intellectual disability
